# Dissecting the Effect of Berberine on the Intestinal Microbiome in the Weaned Piglets by Metagenomic Sequencing

**DOI:** 10.3389/fmicb.2022.862882

**Published:** 2022-04-07

**Authors:** Hong Hu, Kexing Xu, Kunping Wang, Feng Zhang, Xi Bai

**Affiliations:** ^1^College of Animal Science, Anhui Science and Technology University, Chuzhou, China; ^2^Anhui Province Key Laboratory of Animal Nutrition Regulation and Health, Chuzhou, China

**Keywords:** weaned piglets, berberine, gut microbiota, metagenomic sequencing, microbial function

## Abstract

This study aimed to investigate the microbial structure and function in the rectum of weaned piglets with berberine supplementation. Twelve healthy 21-day-old Duorc × (Landrace × Large White) weaned piglets (similar body weight) were evenly divided into control and berberine groups and were fed a basal diet supplemented with 0 and 0.1% berberine, respectively. After 21 days, metagenomic sequencing analysis was performed to detect microbial composition and function in the rectum of weaned piglets. Results showed that there were 10,597,721,931–14,059,392,900 base pairs (bp) and 10,186,558,171–15,859,563,342 bp of clean data in the control and berberine groups, respectively. The Q20s of the control and berberine groups were 97.15 to 97.7% and 96.26 to 97.68%, respectively. The microorganisms in the berberine group had lower (*p* < 0.05) Chao1, alternating conditional expectation, Shannon, and Simpson indices at the species levels than those in the control group. Analysis of similarity showed that there were significant differences (*p* < 0.01) between the control and berberine groups at the genus and species levels of the gut microorganisms. Dietary berberine significantly increased (*p* < 0.05) the abundance of *Subdoligranulum variabile*, but decreased (*p* < 0.05) the abundance of *Prevotella copri* compared with the control group. Carbohydrate-active enzymes analysis revealed that the levels of polysaccharide lyases and carbohydrate esterases were lower (*p* < 0.05) in the berberine group than that in the control group. Linear discriminant analysis effect size analysis showed that berberine supplementation could induce various significant Kyoto Encyclopedia of Genes and Genomes pathways, including carbohydrate metabolism, environmental information processing, and microbial metabolism in diverse environments. In conclusion, our findings suggest that berberine could improve the composition, abundance, structure, and function of gut microbiome in the weaned piglets, potentially providing a suitable approach for the application of berberine in human and animal health.

## Introduction

Berberine is an isoquinoline alkaloid isolated from the traditional Chinese herb *Coptis chinensis*, which is widely used for its medicinal properties. Berberine has antidiarrheal, antibacterial, anti-inflammatory, antitumor, and hypoglycemic effects. It has good therapeutic effect on intestinal inflammation, diabetes, hypertension, and tumors (Fu et al., 2020; Huang et al., 2021; Patel, 2021). Specifically, the therapeutic effect of berberine on intestinal bacterial infection has been investigated for its potential use in clinical practice (Yu M. et al., 2020). Berberine helps in maintaining the intestinal health as it accumulates in the intestine easily and is beneficial in improving the imbalance in intestinal bacteria (Wu et al., 2020).

The intestinal microflora is a complex microbial system composed of a variety of microorganisms participating in numerous physiological processes of the body (Jin et al., 2019; Ma and Ma, 2019; Sun et al., 2020). The intestine harbors various microorganisms such as *Lactobacillus*, *Bacillus*, *Enterobacter*, *Bifidobacterium*, and *Enterococcus*. Initially, the gut microflora was thought to be closely related only to digestion and nutrient absorption; however, recent studies reported that it affects the body health by regulating metabolic diseases, such as obesity, diabetes, and cardiovascular diseases, as well as immune-related disorders (Schippa and Conte, 2014; Yadav and Jha, 2019). As the largest and most complex microecosystem of the body, intestinal microorganisms and their metabolites play an important role in animal health.

Limited studies have focused on the possible link between microbiome and function of gut microflora with berberine supplementation in weaned piglets. With the development of metagenomic high-throughput sequencing technology, it is possible to analyze a large number of microbial community species, abundance, and related biological information by performing total microbial DNA extraction from a specific environment and library construction (Fraher et al., 2012; Zhou et al., 2015; Quan et al., 2019). Thus, a large amount of information on non-culturable microbial flora can be obtained without the need of isolation and culture methods used in traditional microbial research (Guo et al., 2014; Walker et al., 2014). Currently, metagenomic sequencing technology has become an important tool to study intestinal environmental microorganisms. In the present study, this technique was used to characterize the microbial composition and function in the rectum of weaned pigs supplemented with berberine.

## Materials and Methods

### Animals and Experimental Design

The animal experimental design was approved by the Animal Care and Use Committee of Anhui Science and Technology University. Twelve healthy 21-day-old Duorc × (Landrace × Large White) weaned piglets (similar body weight) were purchased from Qingxuan Agricultural Development Co., Ltd. (Bengbu, China), and equally divided into the control and berberine groups (six replicates and one pig/replicate). Pigs in the control and berberine groups were fed a basal diet supplemented with 0 and 0.1% berberine, respectively. Berberine chloride hydrate (purity ≥ 98%) was obtained from Aladdin Reagent Co., Ltd. (China). A basal diet (Table 1) was designed on the basis of National Research Council [NRC] (2012). Piglets could feed and drink water freely.

**TABLE 1:** Composition and nutrient levels of basal diets (%, as-fed basis).

Items	Content (%)
Corn	37.50
Puffed corn	15.00
Soybean meal	15.00
Puffed soybean	10.0
Egg yolk power	2.00
Fish meal	2.50
Whey power	10.00
Sugar	2.00
Soybean oil	2.00
Vitamin and mineral premix*	4.00
Total	100.00
Nutrient levels	
CP	19.15
DE, MJ/kg	14.64
Lys	1.38
Thr	0.86
Met	0.41
Ca	0.65
AP	0.35

*Provided per kilogram of diet: Zn (ZnSO_4_⋅H_2_O), 100 mg; Cu (CuSO_4_⋅5H_2_O), 125 mg; Mn (MnSO_4_⋅H_2_O), 60 mg; Fe (FeSO_4_⋅H_2_O), 120 mg; I [Ca(IO_3_)_2_], 0.6 mg; Se (Na_2_SeO_3_), 0.30 mg; vitamin A, 10,000 IU; vitamin D_3_, 2,500 IU, vitamin 35 IU; vitamin K_3_, 3.0 IU; vitamin B_5_, 40 mg; nicotinic acid, 60 mg; folic acid, 1 mg; biotin, 0.2 mg; vitamin B_6_, 4.0 mg; vitamin B_2_, 7.5 mg; vitamin B_1_, 5.0 mg; vitamin B_12_, 0.08 mg.

### Sample Collection

After 21 days, the stool samples were collected from the rectum of all piglets by rectal massage. These samples were immediately stored in liquid nitrogen (−196°C) for further analysis and metagenomic sequencing.

### Genomic DNA Extraction

Genomic DNA was isolated from stool samples using Magen HiPure Bacterial DNA Kits (Guangzhou, China). The quality of genomic DNA was verified using Qubit Fluorometric Quantification and Nanodrop Spectrophotometers (Thermo Fisher Scientific, Waltham, MA, United States).

### Metagenomic Sequencing Analysis

Metagenomic sequencing analysis was performed as described by Liu et al. (2020). Briefly, 12 metagenomic DNA libraries were constructed using NEBNext™ M Ltra^®^ DNA Library Prep Kit (NEB, Ipswich, MA, United States) for Illumina. Polymerase chain reaction was used to amplify 300- to 400-bp-long DNA fragments. Metagenomic sequencing was carried out on an Illumina Novaseq 6000 platform at Gene *Denovo* Biotechnology Co., Ltd. (Guangzhou, China). Clean data were obtained from raw data using FASTP 18.0 software (Chen et al., 2018), which was used for further genome assembly.

### Bioinformatics Analysis

Bioinformatics analysis of the metagenomic sequence was performed as described by Liu et al. (2020). Gene assembly and prediction were performed using MEGAHIT 11.2 and MetaGeneMark 3.38, respectively.

α Diversity refers to the richness of species/functions in an intestinal microbial environment, which indicates the balance state and living conditions of the gut microorganisms. Analysis of α diversity with Chao1, alternating conditional expectation (ACE), Shannon, and Simpson parameters were performed using the Python scikit-bio package.

Analysis of similarity (ANOSIM) is a test method for analyzing microbial community structure, which is used to test whether the difference between groups is significantly greater than that within groups. ANOSIM test was performed using the vegan R package.

The Venn graph was plotted using VennDiagram package in R project. Welch *t* and analysis of variance (ANOVA) tests were used to show the species with significant differences between the two groups. Prediction of carbohydrate-active enzymes (CAZy) was performed using the CAZy databases. Kyoto Encyclopedia of Genes and Genomes (KEGG) pathway analysis was performed using the DIAMOND software in the KEGG databases. Linear discriminant analysis effect size (LEfSe) analysis was performed by LEfSe software. All bioinformatics analyses were performed using the R software, and *p* < 0.05 indicates statistical significance.

## Results

### Analysis of the Intestinal Microbial Metagenomic Sequencing Data in Weaned Piglets

Twelve metagenomic DNA libraries constructed from the control and berberine groups were sequenced on the Illumina Novaseq 6000 platform. As shown in Table 2, there were 10,622,678,100–14,120,565,000 base pairs (bp) and 10,226,653,500–15,941,277,000 bp of raw data in the control and berberine groups, respectively. After filtering these data, 10,597,721,931–14,059,392,900 bp and 10,186,558,171–15,859,563,34 bp of clean data were obtained in the control and berberine groups, respectively (Table 2). The Q20s (%) of the control and berberine groups were 97.15 to 97.7% and 96.26 to 97.68%, respectively (Table 2). Furthermore, the GC contents (%) of the control and berberine groups were 43.86 to 47.3% and 42.94 to 49.59%, respectively (Table 2). Negligible *n* (%) content was found in both groups (Table 2).

**TABLE 2:** Sequencing data of intestinal microbial metagenomics in weaned piglets.

Sample	Raw data (bp)	Clean data (bp)	Q20 (%)	*n* (%)	GC (%)
Control1	10,814,343,000	10,785,737,116 (99.74%)	10,537,625,000 (97.7%)	288,590 (0.0%)	5,101,805,024 (47.3%)
Control2	10,622,678,100	10,597,721,931 (99.77%)	10,324,690,051 (97.42%)	293,195 (0.0%)	4,895,352,310 (46.2%)
Control3	10,826,112,300	10,795,693,976 (99.72%)	10,527,897,184 (97.52%)	298,263 (0.0%)	5,017,055,625 (46.47%)
Control4	11,016,380,100	10,987,285,740 (99.74%)	10,718,060,574 (97.55%)	260,469 (0.0%)	5,420,474,218 (49.33%)
Control5	11,120,942,100	11,088,979,787 (99.71%)	10,799,013,973 (97.39%)	274,779 (0.0%)	5,113,743,984 (46.12%)
Control6	14,120,565,000	14,059,392,900 (99.57%)	13,659,274,446 (97.15%)	150,051 (0.02%)	6,166,217,653 (43.86%)
Berberine1	10,226,653,500	10,186,558,171 (99.61%)	9,805,479,415 (96.26%)	169,146 (0.0%)	4,636,233,462 (45.52%)
Berberine2	10,891,334,700	10,867,882,674 (99.78%)	10,589,454,821 (97.44%)	276,217 (0.0%)	5,389,587,090 (49.59%)
Berberine3	10,629,873,300	10,609,483,224 (99.81%)	10,349,069,808 (97.55%)	281,753 (0.0%)	4,956,793,040 (46.72%)
Berberine4	10,745,099,400	10,720,204,112 (99.77%)	10,431,619,440 (97.31%)	299,057 (0.0%)	5,289,747,437 (49.34%)
Berberine5	15,941,277,000	15,859,563,342 (99.49%)	15,285,113,751 (96.38%)	169,138 (0.02%)	6,809,305,846 (42.94%)
Berberine6	10,531,396,200	10,507,346,266 (99.78%)	10,263,382,226 (97.68%)	384,034 (0.0%)	4,658,637,700 (44.34%)

*Q20 (%) is the percentage of equal Q20 data in the clean data; n (%) is the percentage of N bases in the clean data. GC (%) is the percentage of G and C bases in clean data.*

### Effect of Berberine on Microbiome Diversity (α-Diversity Analysis) of Pig Gut Microbiome

**FIGURE 1:**
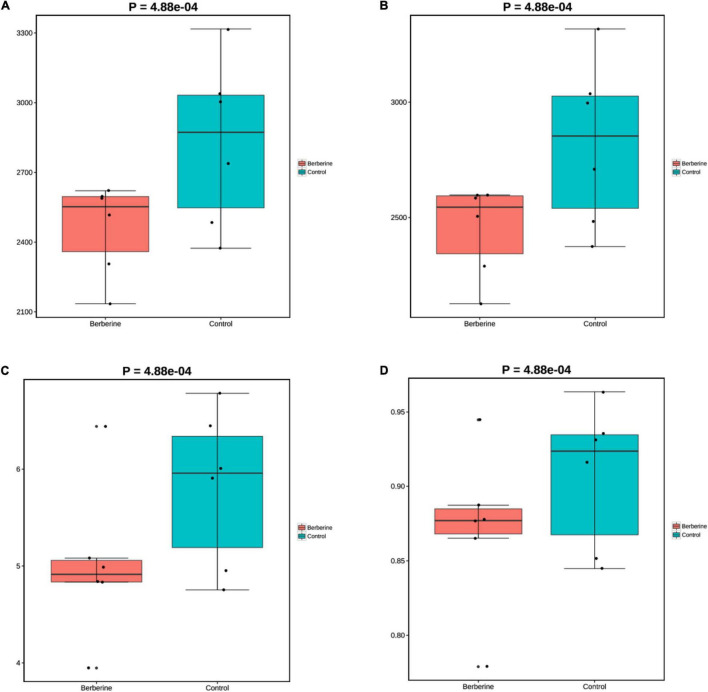
Effects of berberine on microbiome diversity (α-diversity analysis) of pig gut microbiome (**A:** Chao1; **B:** ACE; **C:** Shannon; **D:** Simpson).

The microorganisms in the berberine group had lower (*p* < 0.05) Chao1, ACE, Shannon, and Simpson indices at the species levels than those in the control group (Figure 1).

### Analysis of Similarity Between the Control and Berberine Groups

**FIGURE 2:**
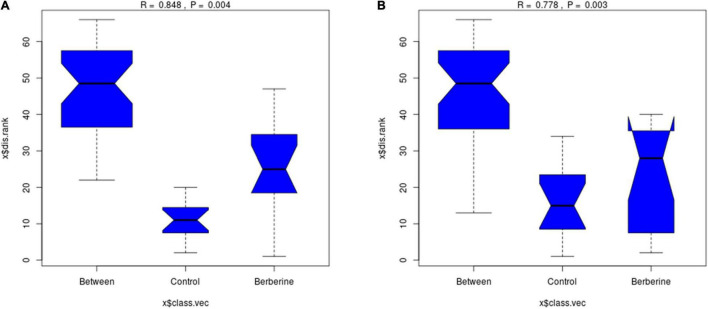
Analysis of similarity analysis between control and berberine groups (**A:** genus levels; **B:** species levels).

As shown in Figure 2, there was significant difference (*p* < 0.01) between the control and berberine groups at the genus and species levels of the gut microorganisms.

### Species Venn Analysis Between the Control and Berberine Groups

**FIGURE 3:**
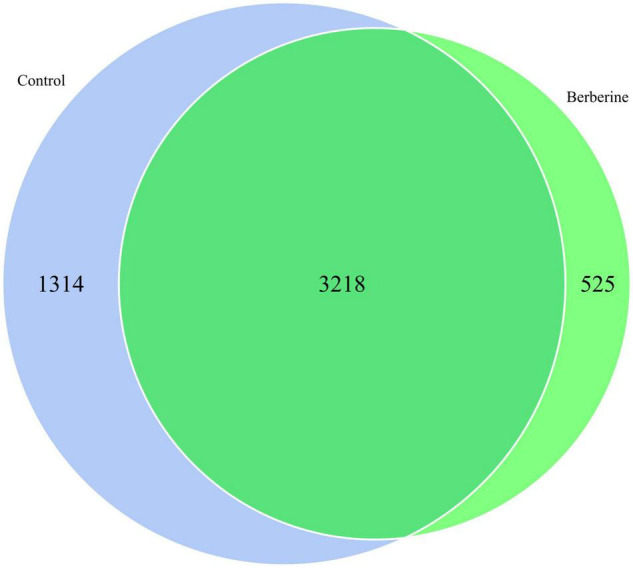
Species Venn analysis between control and berberine groups.

The species distribution in microbial communities of the different treatment groups has a certain degree of similarity and specificity. In order to understand the species differences, Venn diagram was used to show the common and unique information between the different groups based on the species abundance information of samples. As shown in Figure 3, a total of 3,218 microbial species were common in both groups; however, 1,314 and 525 microbial species were unique in the control and berberine groups, respectively.

### Effects of Berberine on Microbial Species of Pig Gut Microbiome

**FIGURE 4:**
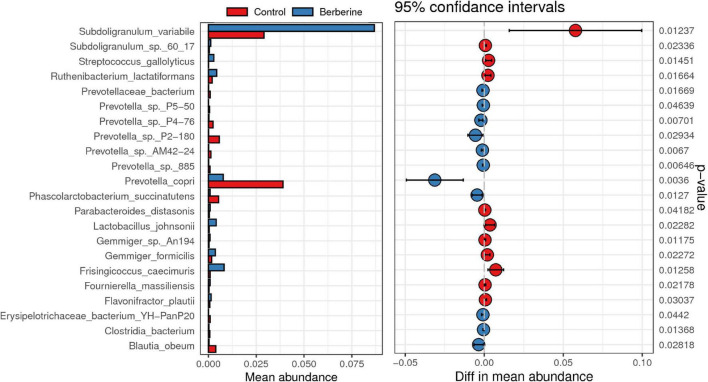
Welch *t*-test of berberine on microbial species of pig gut microbiome.

**FIGURE 5:**
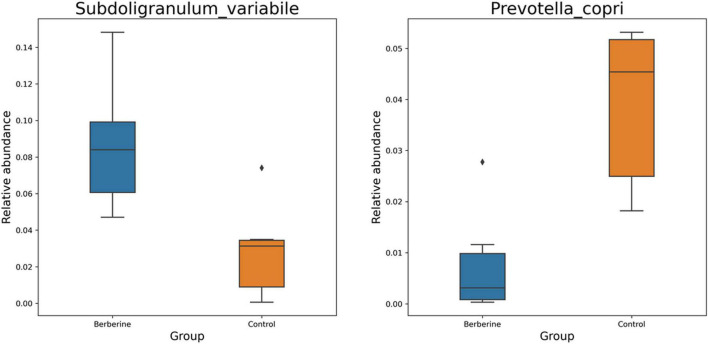
ANOVA test of berberine on microbial species of pig gut microbiome.

Welch *t*-test showed that berberine supplementation significantly increased (*p* < 0.05) the abundance of *Subdoligranulum variabile*, *Lactobacillus johnsonii*, *Parabacteroides distasonis*, *Fournierella massiliensis*, *Ruthenibacterium lactatiformans*, *Frisingicoccus caecimuris*, and *Gemmiger formicilis*, but significantly decreased (*p* < 0.05) the abundance of *Prevotella copri*, *Prevotella* sp. P2-180, *Prevotella* sp. P4-76, *Prevotella* sp. AM42-24, *Prevotella* sp. 885, *Prevotella* sp. P5-50, *Erysipelotrichaceae bacterium* YH-PanP20, *Prevotellaceae bacterium*, and *Phascolarctobacterium succinatutens* compared with the control group (Figure 4). ANOVA test showed that berberine supplementation significantly increased (*p* < 0.05) the abundance of *S. variabile*, but significantly decreased (*p* < 0.05) the abundance of *P. copri* compared with the control group (Figure 5).

### CAZy Analysis Between the Control and Berberine Groups

**FIGURE 6:**
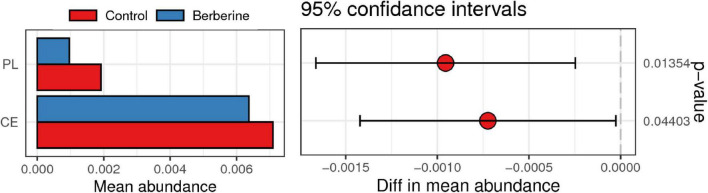
Analysis of CAZy between control and berberine groups.

CAZy include glycoside hydrolases, glycosyl transferases, polysaccharide lyases (PLs), carbohydrate esterases (CEs), and auxiliary activities. As shown in Figure 6, PL and CE levels were lower in the berberine group than that in the control group.

### Kyoto Encyclopedia of Genes and Genomes Analysis Between the Control and Berberine Groups

**FIGURE 7:**
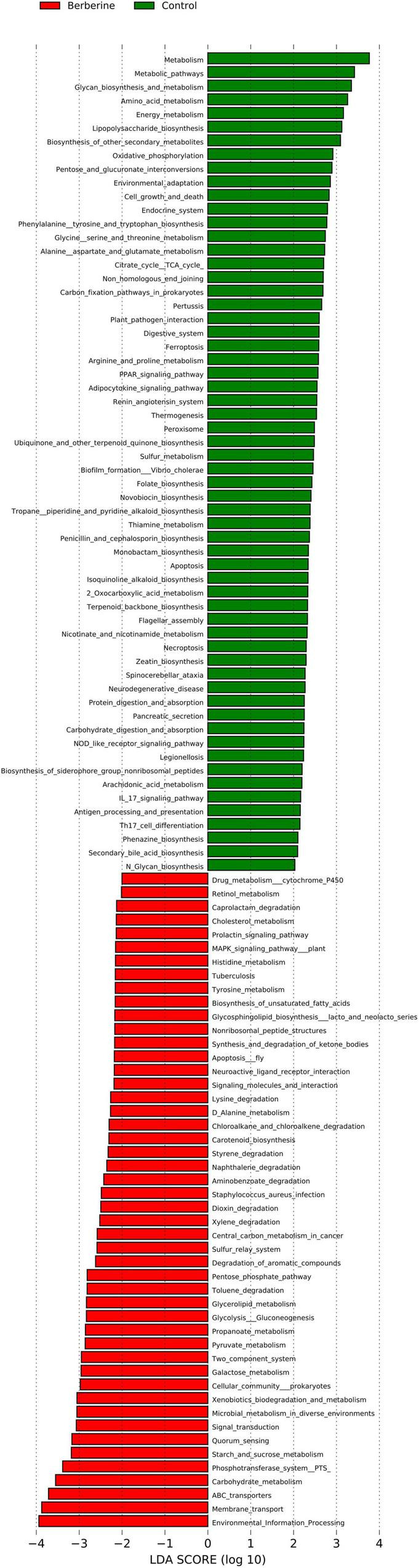
Analysis of KEGG between control and berberine groups.

The results of LEfSe analysis in the berberine group were significantly associated with various KEGG pathways, including carbohydrate metabolism, environmental information processing, microbial metabolism in diverse environments, drug metabolism cytochrome P450, cellular community prokaryotes, dioxin degradation, xylene degradation, *Staphylococcus aureus* infection, starch and sucrose metabolism, toluene degradation, and so on (Figure 7).

## Discussion

Early weaning of piglets can shorten the slaughter cycle of pigs and improve the reproductive performance of sows. However, incomplete development of intestinal microbiota in early weaned piglets may lead to intestinal irritability and reduced production performance (Moeser et al., 2017; Upadhaya and Kim, 2021). Antibiotics can improve the above conditions, but the problem of antibiotic residues needs to be addressed (Yang et al., 2019). Berberine, a traditional Chinese herbal extract, has strong antibacterial effect and is an effective antibiotic substitute (Xu et al., 2020; Zhu et al., 2021). However, few studies have focused on the effect of berberine on the gut microbiota in early weaned piglets. In this work, the macrogenomics and high-throughput sequencing techniques were used to investigate the effects of berberine on intestinal microbiome and function of early weaned piglets (weaning age: 21 days).

Metagenomic analysis involves the DNA extraction from all microorganisms in environmental samples directly followed by a metagenomic library construction and uses high-throughput sequencing technique to study the genetic composition and community functions of these microorganisms (Prayogo et al., 2020). Animal microbial population is distributed on the body surface, oral cavity, gastrointestinal tract, and reproductive tracts, but there are significant differences in the types and quantities of microorganisms based on dietary, species, sex, and age. The microbes in the gut are more numerous than the body’s other organs. The complexity of the environment and microorganisms results in less rigorous data analysis using traditional methods; however, the development of metagenomics and high-throughput sequencing technology has promoted the study of gut microbes considerably (Walker et al., 2014; Guo et al., 2021). In the present study, a major part of raw data (>99%) contributed to the clean data, and the Q20 (%) was more than 96% in the gut microbiomes of the control and berberine groups. A total of 68.7 and 68.3 billion bp clean reads were obtained by conducting metagenomic sequencing of the control and berberine groups, respectively. ANOSIM revealed that the control and berberine groups had significant differences at the genus and species levels of intestinal microorganisms in weaned piglets. These results suggest that the metagenomic sequencing data were reliable and sufficient to investigate the effects of berberine on the gut microbial diversity and function in weaned piglets.

Nutritional digestion and absorption, physiology, metabolism, intestinal barrier, immune function, and disease onset are affected by the composition, diversity, and functional changes in intestinal microflora (Chang and Martinez-Guryn, 2019; Liu et al., 2019). Accordingly, the stability of microecological environment plays a crucial role in animal health regulation. Berberine shows a broad-spectrum antibacterial effect against a variety of gram-positive, gram-negative, and drug-resistant bacteria (Yue et al., 2019; Jamshaid et al., 2020). The effects of berberine influence intestinal infectious disease development and body health through the inhibition of intestinal bacteria (Zhu et al., 2021). Studies have shown that berberine can directly regulate the structure of intestinal microbiota by reducing the number of intestinal microbes in a dose-dependent manner (Zhang et al., 2019). In metagenomics research, Chao1, ACE, Shannon, and Simpson indices are used to study the gut microbiome diversity. We found that dietary 0.1% berberine significantly decreased these indices at the species level compared with those in the control group, suggesting toward the efficacy of berberine in reducing the richness and diversity of intestinal microbiome. Similarly, research conducted by Zhang et al. (2019) revealed that berberine decreased the diversity and quantity of the intestinal microflora in db/db mice.

Berberine is not easily absorbed after oral administration; thus, it can maintain a high concentration in the gastrointestinal tract, providing the necessary conditions required for inhibiting the intestinal bacterial growth (Cheng et al., 2021). In addition, as an antibacterial drug, it can inhibit a variety of pathogenic bacteria and change the structure of intestinal microflora (Habtemariam, 2020; Yu C. et al., 2020; Cheng et al., 2021). The most common mechanism of berberine-mediated regulation of intestinal flora is to change the original dominant intestinal bacteria to maintain the microecological balance. Habtemariam (2020) suggested that the underlying mechanism for the multifunctional role of berberine was its regulation of gut microbiota. Zhang et al. (2012) showed that berberine with high-fat diet in rats could increase the abundance of *Allobaculum* and *Blautia* in the intestine. Dietary berberine also increased the abundance of beneficial bacteria including *S. variabile*, *L. johnsonii*, and *P. distasonis*, as shown in the present study. *S. variabile* improves gut mucosal immune response and inhibits food allergy in mice (Abdel-Gadir et al., 2019). *L. johnsonii* can promote growth, gut development, and intestinal microorganisms in pig, mice, and chicken, when used as a probiotic (Wang et al., 2017; He et al., 2019; Wang et al., 2020). The abundance of *P. distasonis* was negatively correlated with obesity, non-alcoholic fatty liver disease, diabetes, and other disease states, suggesting that it possibly plays a positive regulatory role in glucose and lipid metabolism (Wang et al., 2019). By contrast, dietary berberine decreased the abundance of *P. copri*, which leads to changes in microbiota metabolism and reduces interleukin-18 production. This aggravates the intestinal inflammation and may result in systemic autoimmunity (Ley, 2016).

Berberine can activate some signaling pathways and carbohydrate-related enzymes by improving intestinal microflora and health (Liao et al., 2020; Li et al., 2021). Findings from the present study revealed that berberine changed the structure, abundance, and function of gut microbiota in weaned piglets. Alterations in the gut microbiota lead to functional changes as well. Dietary berberine could markedly affect the CAZy activity of intestinal microflora. Similarly, Li et al. (2021) reported that berberine treatment affects the carbohydrate utilization by altering CAZy activity in the intestinal microflora. Alignment analysis based on KEGG database showed significantly enriched carbohydrate metabolism and environmental information processing pathways in the berberine group. Carbohydrate metabolism pathway mainly involves carbohydrate digestion to provide energy for microbial growth through fermentation in the large intestine, which leads to generation of volatile fatty acids and their derivatives to provide nutrition for the body (Tremaroli and Bäckhed, 2012). Environmental information processing pathway is related to the changes in intestinal microbiota (Arboleya et al., 2016).

## Conclusion

In summary, there were microbial community and functional differences in the rectum of weaned piglets between the control and berberine groups. We demonstrated that berberine could improve the composition, abundance, structure, and function of gut microbiome in the weaned piglets. Our research might provide a novel scientific basis for the further development and application of berberine (such as replacing antibiotics) in the feed and food industries.

## Data Availability Statement

The raw sequencing data presented in the study are deposited in the National Center for Biotechnology Information (NCBI) Sequence Read Archive (SRA) repository, accession number PRJNA807368.

## Ethics Statement

The animal study was reviewed and approved by the Animal Care and Use Committee of Anhui Science and Technology University.

## Author Contributions

HH and XB: data the collection and drafting the manuscript. HH, FZ, and XB: conceive and design the study. KX and KW: statistical analysis. FZ: critical revision of the manuscript. All authors contributed to the article and approved the submitted version.

## Conflict of Interest

The authors declare that the research was conducted in the absence of any commercial or financial relationships that could be construed as a potential conflict of interest.

## Publisher’s Note

All claims expressed in this article are solely those of the authors and do not necessarily represent those of their affiliated organizations, or those of the publisher, the editors and the reviewers. Any product that may be evaluated in this article, or claim that may be made by its manufacturer, is not guaranteed or endorsed by the publisher.

## References

[B1] Abdel-Gadir A. Stephen-Victor E. Gerber G. K. Rivas M. N. Wang S. Harb H. (2019). Microbiota therapy acts via a regulatory T cell MyD88/RORγt pathway to suppress food allergy. *Nat. Med.* 25 1164–1174. 10.1038/s41591-019-0461-z 31235962 PMC6677395

[B2] Arboleya S. Sánchez B. Solís G. Fernández N. Suárez M. Hernández-Barranco A. M. (2016). Impact of prematurity and perinatal antibiotics on the developing intestinal microbiota: a functional inference study. *Int. J. Mol. Sci.* 17:649. 10.3390/ijms17050649 27136545 PMC4881475

[B3] Chang E. B. Martinez-Guryn K. (2019). Small intestinal microbiota: the neglected stepchild needed for fat digestion and absorption. *Gut Microbes* 10 235–240. 10.1080/19490976.2018.1502539 30136893 PMC6546320

[B4] Chen S. Zhou Y. Chen Y. Gu J. (2018). fastp: an ultra-fast all-in-one FASTQ preprocessor. *Bioinformatics* 34 884–890. 10.1093/bioinformatics/bty560 30423086 PMC6129281

[B5] Cheng H. Liu J. Tan Y. Feng W. Peng C. (2021). Interactions between gut microbiota and berberine, a necessary procedure to understand the mechanisms of berberine. *J. Pharm. Anal.* 21, 117–119. 10.1016/j.jpha.2021.10.003 PMC9463479 36105164

[B6] Fraher M. H. O’Toole P. W. Quigley E. M. (2012). Techniques used to characterize the gut microbiota: a guide for the clinician. *Nat. Rev. Gastroenterol. Hepatol.* 9 312–322. 10.1038/nrgastro.2012.44 22450307

[B7] Fu K. Xu M. Zhou Y. Li X. Wang Z. Liu X. (2020). The Satus quo and way forwards on the development of Tibetan medicine and the pharmacological research of Tibetan materia medica. *Pharmacol. Res.* 155:104688. 10.1016/j.phrs.2020.104688 32061838

[B8] Guo L. Zhang D. Fu S. Zhang J. Zhang X. He J. (2021). Metagenomic sequencing analysis of the effects of colistin sulfate on the pig gut microbiome. *Front. Vet. Sci.* 8:663820. 10.3389/fvets.2021.663820 34277753 PMC8282896

[B9] Guo M. Huang K. Chen S. Qi X. He X. Cheng W. H. (2014). Combination of metagenomics and culture-based methods to study the interaction between ochratoxin A and gut microbiota. *Toxicol. Sci.* 141 314–323. 10.1093/toxsci/kfu128 24973096 PMC4833112

[B10] Habtemariam S. (2020). Berberine pharmacology and the gut microbiota: a hidden therapeutic link. *Pharmacol. Res.* 155:104722. 10.1016/j.phrs.2020.104722 32105754

[B11] He T. Zhu Y. H. Yu J. Xia B. Liu X. Yang G. Y. (2019). *Lactobacillus johnsonii* L531 reduces pathogen load and helps maintain short-chain fatty acid levels in the intestines of pigs challenged with *Salmonella enterica* infantis. *Vet. Microbiol.* 230 187–194. 10.1016/j.vetmic.2019.02.003 30827387

[B12] Huang J. Feng W. Li S. Tang H. Qin S. Li W. (2021). Berberine exerts anti-cancer activity by modulating adenosine monophosphate-activated protein kinase (AMPK) and the phosphatidylinositol 3-kinase/protein kinase B (PI3K/AKT) signaling pathways. *Curr. Pharm. Des.* 27 565–574. 10.2174/1381612826666200928155728 32988344

[B13] Jamshaid F. Dai J. Yang L. X. (2020). New development of novel berberine derivatives against bacteria. *Mini Rev. Med. Chem.* 20 716–724. 10.2174/1389557520666200103115124 31902359

[B14] Jin M. Qian Z. Yin J. Xu W. Zhou X. (2019). The role of intestinal microbiota in cardiovascular disease. *J. Cell. Mol. Med.* 23 2343–2350. 10.1111/jcmm.14195 30712327 PMC6433673

[B15] Ley R. E. (2016). *Prevotella* in the gut: choose carefully. *Nat. Rev. Gastroenterol. Hepatol.* 13 69–70. 10.1038/nrgastro.2016.4 26828918

[B16] Li X. Su C. Jiang Z. Yang Y. Zhang Y. Yang M. (2021). Berberine attenuates choline-induced atherosclerosis by inhibiting trimethylamine and trimethylamine-N-oxide production via manipulating the gut microbiome. *NPJ Biofilms Microbe* 7:36. 10.1038/s41522-021-00205-8 33863898 PMC8052457

[B17] Liao Z. Xie Y. Zhou B. Zou B. Xiao D. Liu W. (2020). Berberine ameliorates colonic damage accompanied with the modulation of dysfunctional bacteria and functions in ulcerative colitis rats. *Appl. Microbiol. Biotechnol.* 104 1737–1749. 10.1007/s00253-019-10307-1 31867696

[B18] Liu H. Hu L. Han X. Zhao N. Xu T. Ma L. (2020). Tibetan sheep adapt to plant phenology in alpine meadows by changing rumen microbial community structure and function. *Front. Microbiol.* 11:587558. 10.3389/fmicb.2020.587558 33193243 PMC7649133

[B19] Liu J. Wang H. W. Lin L. Miao C. Y. Zhang Y. Zhou B. H. (2019). Intestinal barrier damage involved in intestinal microflora changes in fluoride-induced mice. *Chemosphere* 234 409–418. 10.1016/j.chemosphere.2019.06.080 31228844

[B20] Ma N. Ma X. (2019). Dietary amino acids and the gut-microbiome-immune axis: physiological metabolism and therapeutic prospects. *Compr. Rev. Food Sci. Food Saf.* 18 221–242. 10.1111/1541-4337.12401 33337014

[B21] Moeser A. J. Pohl C. S. Rajput M. (2017). Weaning stress and gastrointestinal barrier development: implications for lifelong gut health in pigs. *Anim. Nutr.* 3 313–321. 10.1016/j.aninu.2017.06.003 29767141 PMC5941262

[B22] National Research Council [NRC] (2012). *Nutrient Requirements of Swine.* Washington, DC: National Academy Press.

[B23] Patel P. (2021). A bird’s eye view on a therapeutically ‘wonder molecule’: berberine. *Phytomed. Plus* 1:100070. 10.1016/j.phyplu.2021.100070

[B24] Prayogo F. A. Budiharjo A. Kusumaningrum H. P. Wijanarka W. Suprihadi A. Nurhayati N. (2020). Metagenomic applications in exploration and development of novel enzymes from nature: a review. *J. Genet. Eng. Biotechnol.* 18:39. 10.1186/s43141-020-00043-9 32749574 PMC7403272

[B25] Quan J. Cai G. Yang M. Zeng Z. Ding R. Wang X. (2019). Exploring the fecal microbial composition and metagenomic functional capacities associated with feed efficiency in commercial DLY pigs. *Front. Microbiol.* 10:52. 10.3389/fmicb.2019.00052 30761104 PMC6361760

[B26] Schippa S. Conte M. P. (2014). Dysbiotic events in gut microbiota: impact on human health. *Nutrients* 6 5786–5805. 10.3390/nu6125786 25514560 PMC4276999

[B27] Sun M. Ma N. He T. Johnston L. J. Ma X. (2020). Tryptophan (Trp) modulates gut homeostasis via aryl hydrocarbon receptor (AhR). *Crit. Rev. Food Sci.* 60 1760–1768. 10.1080/10408398.2019.1598334 30924357

[B28] Tremaroli V. Bäckhed F. (2012). Functional interactions between the gut microbiota and host metabolism. *Nature* 489 242–249. 10.1038/nature11552 22972297

[B29] Upadhaya S. D. Kim I. H. (2021). The impact of weaning stress on gut health and the mechanistic aspects of several feed additives contributing to improved gut health function in weanling piglets—a review. *Animals* 11:2418. 10.3390/ani11082418 34438875 PMC8388735

[B30] Walker A. W. Duncan S. H. Louis P. Flint H. J. (2014). Phylogeny, culturing, and metagenomics of the human gut microbiota. *Trends. Microbiol.* 22 267–274. 10.1016/j.tim.2014.03.001 24698744

[B31] Wang H. Ni X. Qing X. Zeng D. Luo M. Liu L. (2017). Live probiotic *Lactobacillus johnsonii* BS15 promotes growth performance and lowers fat deposition by improving lipid metabolism, intestinal development, and gut microflora in broilers. *Front. Microbiol.* 8:1073. 10.3389/fmicb.2017.01073 28659893 PMC5466961

[B32] Wang H. Sun Y. Xin J. Zhang T. Sun N. Ni X. (2020). *Lactobacillus johnsonii* BS15 prevents psychological stress–induced memory dysfunction in mice by modulating the gut–brain axis. *Front. Microbiol.* 11:1941. 10.3389/fmicb.2020.01941 32903531 PMC7438410

[B33] Wang K. Liao M. Zhou N. Bao L. Ma K. Zheng Z. (2019). *Parabacteroides distasonis* alleviates obesity and metabolic dysfunctions via production of succinate and secondary bile acids. *Cell Rep.* 26 222–235. 10.1016/j.celrep.2018.12.028 30605678

[B34] Wu M. Yang S. Wang S. Cao Y. Zhao R. Li X. (2020). Effect of berberine on atherosclerosis and gut microbiota modulation and their correlation in high-fat diet-fed ApoE-/- mice. *Front. Pharmacol.* 11:223. 10.3389/fphar.2020.00223 32231564 PMC7083141

[B35] Xu X. Yang C. Chang J. Wang P. Yin Q. Liu C. (2020). Dietary supplementation with compound probiotics and berberine alters piglet production performance and fecal microbiota. *Animals* 10:511. 10.3390/ani10030511 32204369 PMC7142521

[B36] Yadav S. Jha R. (2019). Strategies to modulate the intestinal microbiota and their effects on nutrient utilization, performance, and health of poultry. *J. Anim. Sci. Biotechnol.* 10:2. 10.1186/s40104-018-0310-9 30651986 PMC6332572

[B37] Yang H. Paruch L. Chen X. van Eerde A. Skomedal H. Wang Y. (2019). Antibiotic application and resistance in swine production in China: current situation and future perspectives. *Front. Vet. Sci.* 6:136. 10.3389/fvets.2019.00136 31157244 PMC6533531

[B38] Yu C. Zhang J. Qin Q. Liu J. Xu J. Xu W. (2020). Berberine improved intestinal barrier function by modulating the intestinal microbiota in blunt snout bream (*Megalobrama amblycephala*) under dietary high-fat and high-carbohydrate stress. *Fish Shellfish Immunol.* 102 336–349. 10.1016/j.fsi.2020.04.052 32360278

[B39] Yu M. Jin X. Liang C. Bu F. Pan D. He Q. (2020). Berberine for diarrhea in children and adults: a systematic review and meta-analysis. *Therap. Adv. Gastroenterol.* 13:1756284820961299. 10.1177/1756284820961299 33149763 PMC7586028

[B40] Yue M. Tao Y. Fang Y. Lian X. Zhang Q. Xia Y. (2019). The gut microbiota modulator berberine ameliorates collagen-induced arthritis in rats by facilitating the generation of butyrate and adjusting the intestinal hypoxia and nitrate supply. *FASEB J.* 33 12311–12323. 10.1096/fj.201900425RR 31425655 PMC6902671

[B41] Zhang W. Xu J. H. Yu T. Chen Q. K. (2019). Effects of berberine and metformin on intestinal inflammation and gut microbiome composition in db/db mice. *Biomed. Pharmacother.* 118:109131. 10.1016/j.biopha.2019.109131 31545226

[B42] Zhang X. Zhao Y. Zhang M. Pang X. Xu J. Kang C. (2012). Structural changes of gut microbiota during berberine-mediated prevention of obesity and insulin resistance in high-fat diet-fed rats. *PLoS One* 7:e42529. 10.1371/journal.pone.0042529 22880019 PMC3411811

[B43] Zhou J. He Z. Yang Y. Deng Y. Tringe S. G. Alvarez-Cohen L. (2015). High-throughput metagenomic technologies for complex microbial community analysis: open and closed formats. *mBio* 6:e02288-14. 10.1128/mBio.02288-14 25626903 PMC4324309

[B44] Zhu C. Huang K. Bai Y. Feng X. Gong L. Wei C. (2021). Dietary supplementation with berberine improves growth performance and modulates the composition and function of cecal microbiota in yellow-feathered broilers. *Poult. Sci.* 100 1034–1048. 10.1016/j.psj.2020.10.071 33518062 PMC7858044

